# Refined Zoning of Landslide Susceptibility: A Case Study in Enshi County, Hubei, China

**DOI:** 10.3390/ijerph19159412

**Published:** 2022-08-01

**Authors:** Zhiye Wang, Chuanming Ma, Yang Qiu, Hanxiang Xiong, Minghong Li

**Affiliations:** College of Environmental Studies, China University of Geosciences, Wuhan 430074, China; wangzhiye@cug.edu.cn (Z.W.); qiuyang@cug.edu.cn (Y.Q.); hanxiangxiong@cug.edu.cn (H.X.); liminghong98@cug.edu.cn (M.L.)

**Keywords:** landslide susceptibility, multi-model, time periods, integrated susceptibility

## Abstract

At present, landslide susceptibility assessment (LSA) based on the characteristics of landslides in different areas is an effective prevention measure for landslide management. In Enshi County, China, the landslides are mainly triggered by high-intensity rainfall, which causes a large number of casualties and economic losses every year. In order to effectively control the landslide occurrence in Enshi County and mitigate the damages caused by the landslide. In this study, eight indicators were selected as assessment indicators for LSA in Enshi County. The analytic hierarchy process (AHP) model, information value (IV) model and analytic hierarchy process-information value (AHP-IV) model were, respectively, applied to assess the landslide distribution of landslides in the rainy season (RS) and non-rainy season (NRS). Based on the three models, the study area was classified into five levels of landslide susceptibility, including very high susceptibility, high susceptibility, medium susceptibility, low susceptibility, and very low susceptibility. The receiver operating characteristic (ROC) curve was applied to verify the model accuracy. The results showed that the AHP-IV model (ROC = 0.7716) was more suitable in RS, and the IV model (ROC = 0.8237) was the most appropriate model in NRS. Finally, combined with the results of landslide susceptibility in RS and NRS, an integrated landslide susceptibility map was proposed, involving year-round high susceptibility, RS high susceptibility, NRS high susceptibility and year-round low susceptibility. The integrated landslide susceptibility results provide a more detailed division in terms of the different time periods in a year, which is beneficial for the government to efficiently allocate landslide management funds and propose effective landslide management strategies. Additionally, the focused arrangement of monitoring works in landslide-prone areas enable collect landslide information efficiently, which is helpful for the subsequent landslide preventive management.

## 1. Introduction

Landslide is one of the most frequent and serious geological hazards entire the globe [1,2,3], and rainfall is an important factor that affects the occurrence of landslides. Landslide susceptibility is an estimate to show “where” the landslides may occur in an area, which is strongly influenced by regional characteristics, especially in the frequency and intensity of rainfall [4,5]. Currently, a range of studies around the world has assessed the susceptibility of landslides with the consideration of rainfall. For example, Robert Emberson [6] et al. systematically analyzed the relationship between landslides and topographic features by processing a list of 16 landslides triggered by large global rainfall events. S. Modugno et al. [7] selected rainfall as an important hazard factor to discuss the monthly global population landslide exposure risk at multiple scales. Additionally, since rainfall can significantly increase the frequency of landslides on the D-658 highway, Kerem Hepdeniz [8] et al. selected rainfall as an indicator to assess the landslide susceptibility of the region, which provide effective landslide management strategies for the managers and stakeholders. Shu-Rong Yang [9] used a discriminant analysis approach to map landslide susceptibility in predicting rainfall-induced landslides in central Taiwan, and Bappaditya Koley [10] et al. used geospatial techniques to analyze the spatial distribution of rainfall-induced landslides along North Sikkim Road Corridor in Sikkim Himalayas, India. Given that rainfall plays a significant role in landslide occurrence, most studies considered rainfall as an assessment indicator and ignored the interrelations between rainfall and landslide.

In Enshi County, Hubei Province, China, the frequent landslide hazards have seriously affected the local economic and social development because of complex geological and environmental conditions and high-intensity rainfall [11,12,13]. As a typical non-engineering measure, LSA is an effective method for landslide damage mitigation, prevention and management [14]. In this study area, the vast literature studied the landslide analysis from different aspects, and many models have been proposed. For example, Xutao Wang [15] et al. made a comprehensive analysis of the stability of the landslide in Enshi County from the perspective of the geological environment and landslide formation mechanism. Bin Zeng [16] et al. analyzed the damage mechanism in the Silurian stratum and used an artificial neural network to establish the spatial distribution of unstable slopes prone to landslides in Enshi County. Wuwen Yan [17] et al. used the rough sets—neural network model to predict the landslide susceptibility in Enshi County by analyzing the formation mechanism. Linfeng Fan [18] et al. applied a weighted information value model to study the landslide susceptibility of Enshi County by using the indicators such as topography, fault, river and lithology. Additionally, Fasheng Miao [19] et al. developed an effective snowmelt model based on severe freezing snow to analyze the landslide susceptibility in Enshi area, etc. By reviewing the previous studies and analyzing the occurrence periods of landslides in previous years, it was found that the landslides in Enshi County mainly occurred in the rainy season (RS), which is significantly higher than the landslides that occurred in the non-rainy season (NRS). Moreover, with the aggravation of global warming and climate change, extreme rainfall in the future is more likely to induce a greater risk of landslide occurrence [20]. Although the current studies pointed out the different landslide distributions in RS and NRS [11,13,21,22,23], there are very rarely studies conducted on landslide susceptibility assessment (LSA) in different periods by consideration of RS and NRS. On a global scale, few studies have assessed landslide susceptibility with the consideration of landslide occurrence in RS and NRS separately.

Currently, scholars use a variety of models to study landslide susceptibility, mainly including qualitative and quantitative models. The qualitative method is an expert-based technique with subjectivity issues in the rating of weights for assessment indicators, while the quantitative method uses mathematical expressions in landslide susceptibility analysis [8,14]. In these models, the analytic hierarchy process (AHP) and information value (IV) models have been widely used and praised [3,24,25,26,27,28,29]. Specifically, AHP is a qualitative and relatively subjective model, which enables well utilization of the advantages of expert experience in classifying and weighting indicators [30]. IV is a quantitative and objective model by a variety of numerical calculations [31]. However, the disadvantage of applying a single model in LSA is also obvious, and relevant studies have shown that the hybrid application of models can significantly improve the accuracy and reliability of the results [32].

Therefore, based on the analysis of landslides data in Enshi County, this study classified the landslides into two categories: RS landslides and NRS landslides, according to the time of landslides occurrence. Additionally, in order to obtain more suitable LSA results, two single models (AHP model and IV model) and a hybrid model (AHP-IV mode) are selected to assess the landslide susceptibility. By assessing the landslide susceptibility at different time periods, this study is able to make the regional landslide susceptibility more accurate in time, which is more helpful for the government to target landslide damage mitigation, prevention and management.

## 2. Materials

### 2.1. Study Area

**Figure 1:**
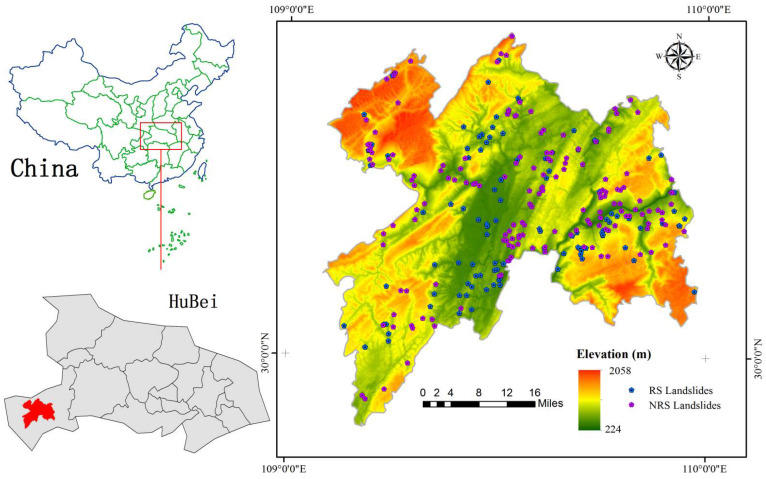
Study area.

The study area Enshi County is located in the southwestern part of Hubei Province, China, with an area of 3976 km^2^, 108°23′12″–110°38′08″ E and 29°07′10″–31°24′13″ N (Figure 1). A total of 273 landslide points were selected in this study, including 108 RS landslides and 165 NRS landslides. The location date and other information about the landslides are shown in the Supplementary Materials.

### 2.2. Technical Route

**Figure 2:**
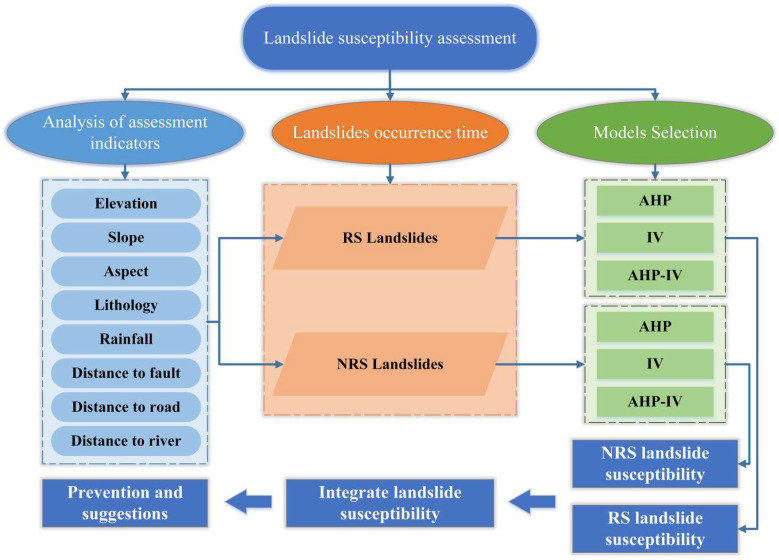
Technical route.

Landslide susceptibility in this study was mapped from two aspects: RS landslides and NRS landslides. AHP, IV and AHP-IV models were, respectively, applied to evaluate the two situations to obtain the final integrated results (Figure 2).

### 2.3. Assessment Indicators

The occurrence of landslides is caused by many factors with a complex causing mechanism. In order to study the connection between landslides distribution and assessment indicators, it is very important to map the assessment indicators partition [33]. Unfortunately, there was no definite specification for the selection and classification of indicators to date [8,14,34]. It is a good way to select suitable indicators for landslide susceptibility according to the local site characteristics [14,24]. Through the field surveys and analysis of the relevant literature, the indicators were divided into two main groups such as internal and trigger indicators [14,35]. Internal indicators include elevation, slope, aspect and lithology. Trigger indicators include distance to fault, distance to the river, distance to road and rainfall. Each indicator vector format layer was converted to raster format with a resolution of 30 × 30 m.

**Figure 3:**
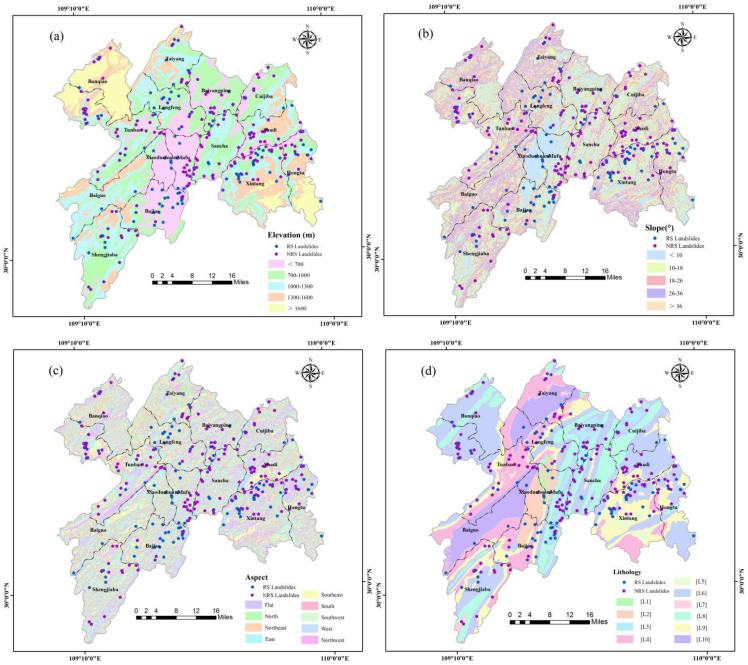

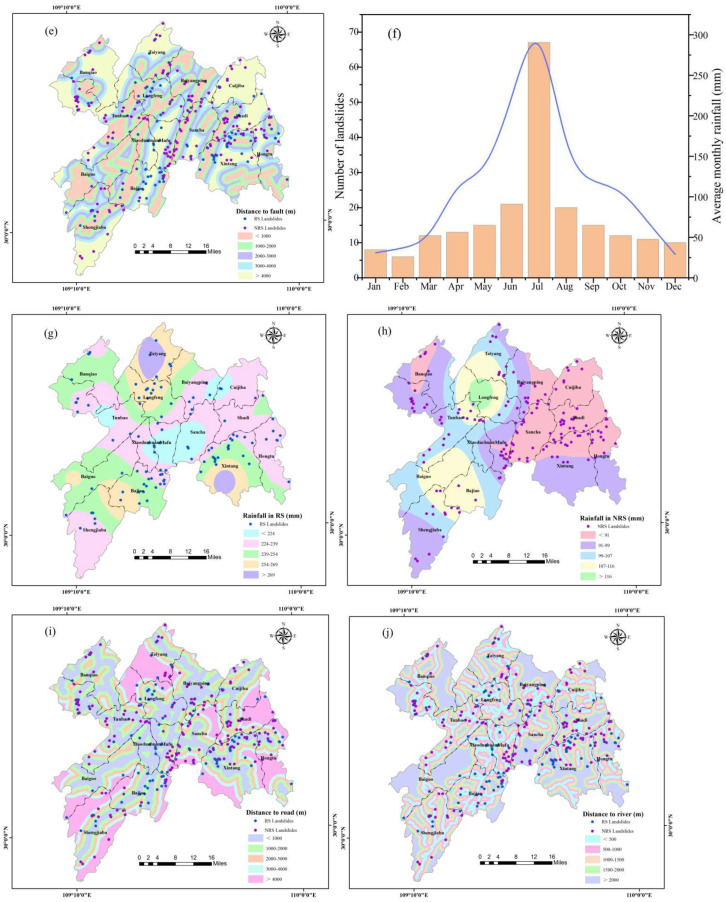
Assessment indicators partition in the study area: (**a**) elevation; (**b**) slope; (**c**) aspect; (**d**) lithology; (**e**) distance to fault; (**f**) plot of the number of landslides versus time; (**g**) rainfall in RS; (**h**) rainfall in NRS; (**i**) distance to road; (**j**) distance to river.

Elevation (E): Elevation is an inherent assessment indicator of landslide occurrence [36]. The study area is a hilly area with a maximum elevation difference of 1834 m. Since many indicators are changing with the dramatic ups and downs in the study area, the elevation becomes an indispensable indicator affecting the occurrence of landslides. In ArcGIS 10.5, elevation was divided into five ranges by natural breaks: <700 m, 700–1000 m, 1000–1300 m, 1300–1600 m and >1600 m (Figure 3a). The elevation data were extracted from Geospatial Data Cloud. Available online: http://www.gscloud.cn (accessed on 12 March 2022).

Slope (S): Slope is one of the main indicators affecting stability [37,38,39]. The slope determines the force distribution on the slope body. With the change in slope, the flow and infiltration of water and the condition of slope deposits have different degrees of influence, thus affecting the overall stability [40]. In ArcGIS 10.5, the slope was divided into five ranges by natural breaks: <10°, 10–18°, 18–26°, 26–36° and >36° (Figure 3b). The slope data were extracted from Geospatial Data Cloud. Available online: http://www.gscloud.cn (accessed on 12 March 2022).

Aspect (A): Aspect is one of the important driving indicators of landslides [21,41]. As the aspect is exposed to different conditions of sunlight, precipitation and weathering, the cohesion of the slope body produces more and more obvious differences under different aspects, thus affecting the overall stability [42]. The aspect was classified into nine ranges: flat, north, northeast, east, southeast, south, southwest, west and northwest (Figure 3c). The aspect data were extracted from Geospatial Data Cloud. Available online: http://www.gscloud.cn (accessed on 12 March 2022).

Lithology (L): The lithology is the material basis of landslides, and different lithology properties have different degrees of influence on landslides occurrence [17,40]. The study area is widely distributed with limestone, sandstone, mudstone and shale with different lithologies, which provide different contributions to landslide occurrence [43]. The lithologies in the study area were classified into ten categories based on different combinations of characteristics (Figure 3d), and the specific classification information is shown in Table 1. The lithology data were provided by the National Geological Archives of China. Available online: https://www.ngac.cn (accessed on 22 April 2022).

Distance to fault (DF): Fault plays an important role in controlling the occurrence of landslides and closely influence the distribution of landslides [17,23]. The formation and development stages of faults have prepared sufficient conditions for the occurrence of landslides in the study area. The developed fissures and fractured rocks increase the probability of slope deformation and damage. The field survey found that most landslides occurred within 1000 m from the fault, and the related studies also used this range to delineate and verify the feasibility [39,44,45,46,47]. In ArcGIS 10.5, the Euclidean distance method was used to classify the distance into five ranges: <1000 m, 1000–2000 m, 2000–3000 m, 3000–4000 m and >4000 m (Figure 3e). The fault data were provided by the National Geological Archives of China. Available online: https://www.ngac.cn (accessed on 22 April 2022).

Rainfall (R): Rainfall is the most important triggering factor of landslides in the study area [12,13,40]. The study area has a humid subtropical monsoon climate with abundant rainfall, which is mostly concentrated in RS from June to August every year. Therefore, we defined June to August as the RS and September to May as the NRS in the study area. Through the statistical analysis of landslide occurrence by month, it was found that although the RS was only three months long, the number of landslides occurred by more than 50% (Figure 3f). The frequency of landslides showed a significant difference from NRS. The multi-year average rainfall distribution in RS and NRS was obtained by using the kriging method and was divided into five ranges by natural breaks in ArcGIS 10.5 (Figure 3g,h). The rainfall data were provided by the Hydrology and Water Resources Survey Bureau of Enshi Autonomous Prefecture, Hubei Province. Available online: http://www.esswj.com (accessed on 25 April 2022).

Distance to road (DRo): Road is a key indicator to reflect the intensity of human engineering activities in landslide susceptibility mapping [30]. The study area is a hilly area, and many public infrastructures have been built on the mountains with little consideration of the surrounding geological environment, thus making the annex area prone to landslides. Through the field survey, it was found that most landslides occurred within 1000 m from the road, and the distance was classified with reference to the relevant literature [8,11,47]. In ArcGIS 10.5, the distance was classified into five ranges: <1000 m, 1000–2000 m, 2000–3000 m, 3000–4000 m and >4000 m using the Euclidean distance method (Figure 3i). The road data were provided by National Catalogue Service For Geographic Information. Available online: https://www.webmap.cn (accessed on 27 April 2022).

Distance to river (Dri): The occurrence of landslides is also closely related to the river [48]. The study area has a dense river network with typical mountainous river characteristics. Due to the continuous scouring of rivers, high and steep air fronts are formed, which provide sliding space for landslides. In addition, landslides are also very likely to occur under the softening effect of water infiltration. The field survey found that most landslides occurred near 500 m from the river, and the distance was classified with reference to the relevant literature [13]. In ArcGIS 10.5, using the Euclidean distance method, the distance to the river was classified into five ranges: <500 m, 500–1000 m, 1000–1500 m, 1500–2000 m and >2000 m (Figure 3j). The river data were provided by National Catalogue Service For Geographic Information. Available online: https://www.webmap.cn (accessed on 27 April 2022).

## 3. Methods

### 3.1. AHP Model

AHP is a semi-quantitative approach that combines the relative importance of each indicator [49]. AHP usually consists of three steps:

Step 1. By comparing each two assessment indicators, they are classified into different importance levels for the occurrence of landslides. The importance of assessment indicators was determined by the 1–9 scale method, with the importance increasing gradually from 1 to 9 (Table 2), and the judgment matrix was constructed;

Step 2. Calculate the maximum eigenvector of the judgment matrix;

Step 3. The random consistency ratio (*CR*) of the judgment matrix was tested, and the calculation of *CR* is shown in Formulas (1) and (2). The constructed judgment matrix is considered valid when *CR* < 0.1.

(1) $$CI=\left(\lambda_{\max}-m\right)/\left(m-1\right)$$

(2) CR=CI/RI

where *CI* is the consistency index of the judgment matrix; *λ*_max_ is the maximum eigenvalue of the judgment matrix; *m* is the order of the judgment matrix; *RI* is the average random consistency index of the judgment matrix.

Thus, the judgment matrix of the importance of assessment indicators (Table 3), the judgment matrix of the weight of assessment indicators during RNS (Table 4) and the judgment matrix of the weight of assessment indicators during RS were obtained (Table 5). Through the consistency test that all the judgment matrices meet the consistency requirements.

The landslide susceptibility index (*LSI*) was calculated by the following Equation:

(3) $$LSI_{\left(AHP\right)}=\sum_{i=1}^{n}W_{i}P_{i}$$

where *LSI* is the landslide susceptibility index; *W_i_* is the weight of each assessment indicator; *P_i_* is the importance of each assessment indicator.

### 3.2. IV Model

IV model is a quantitative analysis method based on the information of each indicator at existing landslides, and the information quantity is used to indicate the possibility of landslides [31]. According to the IV model, the information quantity of the study area is calculated (Table 6). The Equation is as follows:

(4) $$I_{ij}=\ln\frac{N_{j}/N}{S_{j}/S}$$

where *I_ij_* is the information quantity; *N_j_* is the number of grids classified as class *j*; *N* is the total number of grids where landslides occurred; *S_j_* is the number of grids classified as class *j*; *S* is the total number of grids.

The *LSI* was calculated by the following Equation:

(5) $$LSI_{\left(IV\right)}=\sum_{i=1}^{n}I_{ij}$$

where *LSI* is the landslide susceptibility index, and *I_ij_* is the value of information quantity.

### 3.3. AHP-IV Model

Related studies showed that the integrated application of different models helps to improve the accuracy of landslide susceptibility [32]. This assessment combines AHP and IV to construct the AHP-IV model. The specific equation is as follows:

(6) $$LSI_{\left(AHP\text{-}IV\right)}=\sum_{i-1}^{n}W_{i}\times I_{ij}$$

where *LSI* is the landslide susceptibility index; *W_i_* is the weight of each assessment indicator; *I_ij_* is the value of information quantity. 

## 4. Results and Analysis

### 4.1. Landslide Susceptibility Map

**Figure 4:**
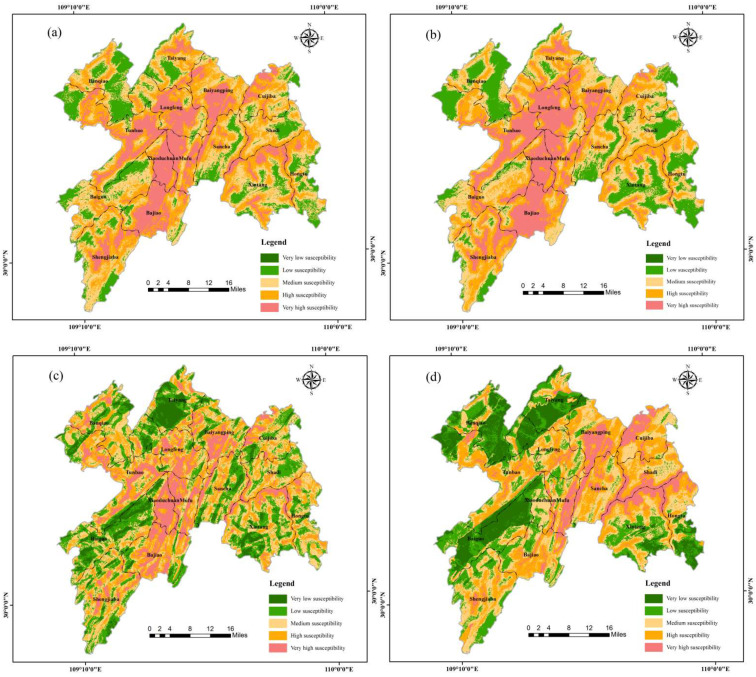

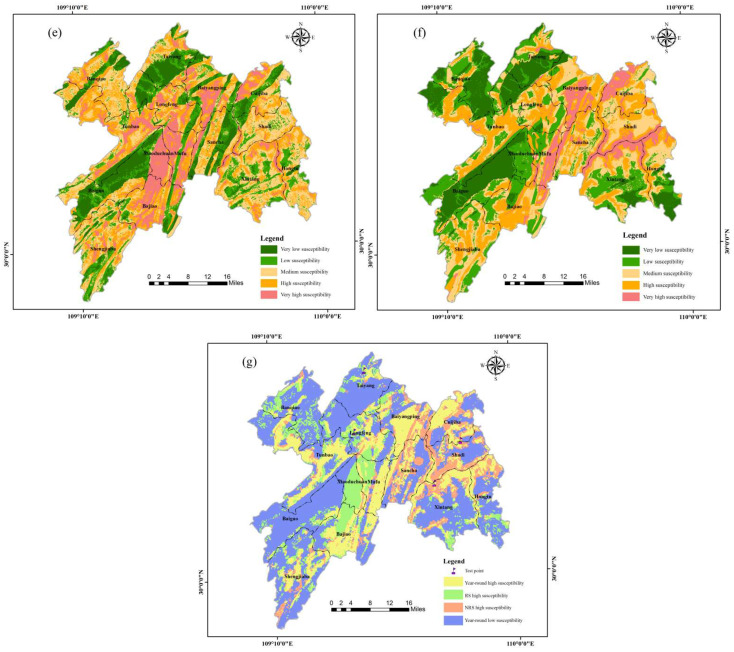
Landslide susceptibility zoning map: (**a**) AHP model in RS; (**b**) AHP model in NRS; (**c**) IV model in RS; (**d**) IV model in NRS; (**e**) AHP-IV model in RS; (**f**) AHP-IV model in NRS; (**g**) Integrate landslide susceptibility zoning map.

Based on the LSI results of each model, the study area was divided into very high susceptibility, high susceptibility, medium susceptibility, low susceptibility and very low susceptibility, as shown in Figure 4a–f.

### 4.2. Model Validation

**Figure 5:**
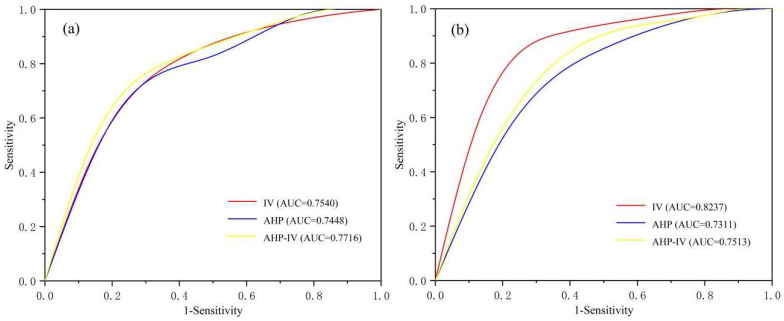
ROC curve of LSA: (**a**) Models in RS; (**b**) Models in NRS.

The receiver operating characteristic (ROC) curve can be used to quantitatively analyze the accuracy of the assessment model [51,52]. The area under the curve (AUC) value is between 0 and 1. The larger the AUC value, the more accurate the corresponding assessment model. When evaluating models for predicting the occurrence of geo-environmental problems, an AUC value of less than 0.5 indicates that the assessment model is inaccurate [53]. The AUC values of the assessment for the AHP, IV and AHP-IV models during RS were 0.7448, 0.7540 and 0.7716, respectively. The AUC values of the assessment for the AHP, IV and AHP-IV models during NRS were 0.7311, 0.8237 and 0.7531, respectively. It showed that all models have a good performance in prediction. Specifically, the AHP-IV model has higher accuracy (AUC = 0.7716) than AHP and IV model in RS (Figure 5a), and the IV model has higher accuracy (AUC = 0.8237) than AHP and AHP-IV model in NRS (Figure 5b). It indicates that the AHP-IV model is more applicable in RS, and the IV model is more applicable in NRS in the study area. The differences in the accuracy of the model can be explained by the complex rainfall effects on the landslide occurrence. The empirical-based AHP method effectively overcame the complex mechanism of rainfall effects and obtained a higher model performance. In fact, the AHP model can correct the unreasonable calculation results (e.g., rainfall contributions on landslides) in the IV model through expert experience, thus improving the model accuracy. Additionally, due to the sparse rainfall in NRS, the influence of rainfall on other assessment indicators is not as great as in RS, so the data-driven model enables obtaining a higher model performance.

### 4.3. Integrate Landslide Susceptibility

Based on the validation results of ROC curves, we selected the landslide susceptibility zoning map with higher accuracy in NRS (Figure 4d) and RS (Figure 4e) to conduct an integrated analysis of the year-round susceptibility of the study area. According to the principle of map integration (Table 7), an integrated zoning principle for landslide susceptibility was first proposed, which divided the study area into year-round high susceptibility, RS high susceptibility, NRS high susceptibility and year-round low susceptibility (Figure 4g). The integrated landslide susceptibility zoning map further demonstrates the RS and NRS landslide susceptibility in different regions and refines the degree of landslide susceptibility in time, and the temporal refinement can enable the government to monitor landslide-prone areas in different time periods in a more targeted manner and help improve the accuracy of landslides monitoring. At the same time, it can also take preventive and control measures in advance for landslide-prone areas in different time periods, which is of great practical significance.

## 5. Discussion

### 5.1. Distribution of Landslide Susceptibility 

According to the landslide susceptibility zoning map (Figure 4d), the very high and high susceptibility areas in NRS are mainly distributed in the central-eastern part, within the area of Baiyangping–Bajiao–Xintang–Cuijiaba. It was found that the faults are widely developed in these areas, which reflects the high contributions of faults to landslide occurrence [12,21]. The lithology in the region is mainly layered by clastic rocks and carbonate rocks with soft and hard interlayers. In these areas, the lithologies are relatively soft and weak, which is more susceptible to landslides. Meanwhile, river scours and soaks increase porewater pressure in the rocks, which leads to a decrease in their shear strength and loss of slope stability. The fragile geological environment is the basis of landslides development [40]. Under the influence of external indicators such as rainfall and river, landslides are more likely to occur in the region. On the other hand, the population density is high, and human engineering activities are intensive, thus changing the surrounding geological environments and affecting the occurrence of landslide disasters.

The medium susceptibility in NRS is mainly distributed in the southwest and northeast of the study area, within the area of Shengjiaba–Sancha–Shadi. In these areas, the elevation of the area is mostly below 1000 m, the slope is less than 26 degrees, and the road construction density is high. Moreover, the lithology is dominated by mudstone and sandstone; the structure is relatively developed, the bedrock is broken and incomplete, and the degree of weathering is high. Landslides are mostly located near roads and rivers.

From the landslide susceptibility zoning map (Figure 4e), areas of very high and high susceptibility in RS are mainly distributed in the central part, within the area of Cuijiaba–Baiyangping–Sancha–Bajiao. It was observed that high and very high susceptibility is more concentrated in the central valley in RS. The main reason for this difference is that the central part is mainly developed as a soft rock body with soft and hard interlayers such as sandstone and shale, which are easily softened by water. The infiltration of prolonged rainfall significantly intensifies the softening effect of lithology, and the increase in precipitation also leads to the enhancement of erosion of slopes by rivers, which results in a significant increase in landslide susceptibility. Combined with the trend of information quantity of assessment indicators (Table 6), it was observed that the information quantity below 18° during RS decreases significantly, while those above 18° increase significantly, which indicates that the rainfall has a greater impact on areas with a higher slope. Likewise, a significant increase within 1000 m of the road was observed, indicating that the long-term rainfall amplifies the impact of human engineering activities on landslides. Moreover, by observing the locations of landslides near the road, it can be found that some areas with better geological conditions also had multiple landslides, further reflecting the influence of human activities on the occurrence of landslides. Therefore, rainfall and human engineering activities are the main triggering indicators for landslides in the study area.

The medium susceptibility in RS is mainly located near Banqiao, Shengjiaba, Shadi, Xinang and Hongtu areas. The elevation in the area is large, the slope is within 26 degrees, and the road construction density is high. The fault is relatively developed; the lithology is mainly tuff, sandstone and shale. The rock layer is relatively broken and incomplete, weathering is strong, and fissures are developed. Landslides are mostly located near roads and rivers, and weak bedrock is prone to landslides under the action of other factors such as rainfall.

### 5.2. Analysis of Assessment Indicators

**Figure 6:**
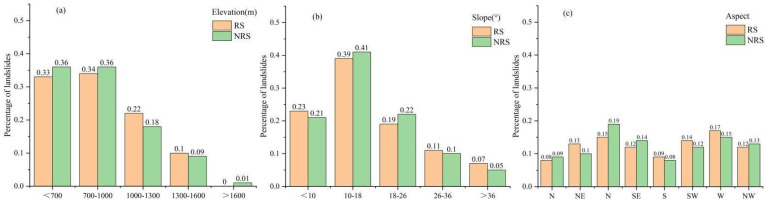

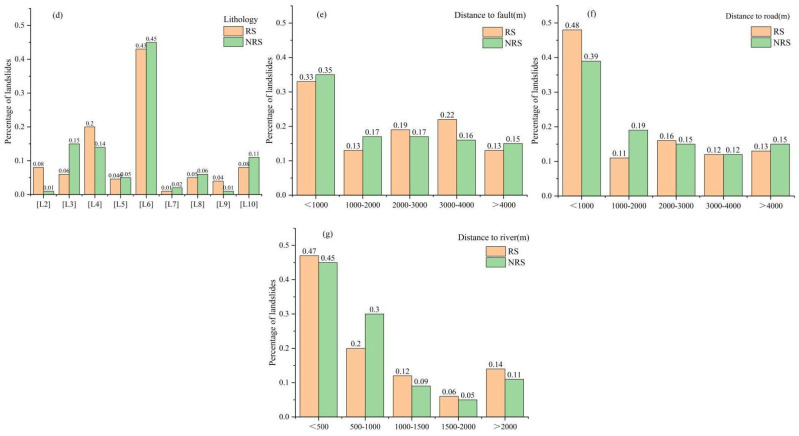
Data of assessment indicators in RS and NRS: (**a**) elevation; (**b**) slope; (**c**) aspect; (**d**) lithology; (**e**) distance to fault; (**f**) distance to road; (**g**) distance to river.

Topography plays an important role in controlling geological hazards and determines the spatial distribution of geological hazard susceptibility to a large extent [36]. The study area is mainly mountainous, with a large difference in elevation between mountains and plains. Under the erosional downcutting of rivers, many numbers of high and steep slopes and ravines have been formed, which provides prerequisites for the occurrence of landslides [17]. As shown in Figure 6a, the percentage of landslides is negatively correlated with altitude, and the lower the altitude, the higher the landslide density. Because of the low terrain and abundant water supply, it is a densely populated area, so there are more human engineering activities, and landslides occur frequently. Landslides are less likely to occur at high altitudes because of the low level of human activity.

Different slope locations have different topographic features, resulting in large differences in overlying rock and gravitational potential energy [54]. From Figure 6b, it can be seen that the percentage of landslides showed a trend of increasing and then decreasing, and landslides mostly occur in areas with lower slopes, mainly concentrated within 26 degrees. Slope plays a significant role in controlling surface water runoff and the accumulation of loose material on slopes [55]. The percentage of landslides occurring under different slope conditions in RS and NRS are approximately the same, indicating that the slope of the study area has a strong controlling effect on the occurrence of landslides.

The aspect has different characteristics under different hydrothermal conditions, which indirectly affects the development of geological hazards [56]. The study area is located in the northern hemisphere. The south, southwest, west and northwest directions have longer sunshine hours and lower soil water content, while the northeast, east, north and southeast directions have shorter sunshine hours, higher soil water content and poor soil stability [42]. It can be seen from Figure 6c that the percentage of landslides occurring on different aspects was not significantly different. The percentage of landslides in the north in NRS is relatively high. In RS, the percentage of landslides in the west is higher, indicating that the abundant rainfall in RS has a certain impact on the aspect, thereby increasing the percentage of landslides.

The composition of lithology and its physicochemical properties have an important influence on the occurrence of geological hazards. As can be seen from Figure 6d, the percentage of landslides in the study area varies widely among lithologies, with landslides mainly occurring in [L6], and the percentage of landslides exceeds 40%. [L6] is widely distributed in the study area and is mainly limestone and shales with thin thickness. It is also weak against weathering and erosion, has poor mechanical properties such as shear strength and is prone to landslides. The results of this assessment showed that landslides are concentrated in both RS and NRS at [L6]. Indicating that lithology is an important controlling factor for landslides in the study area.

In the study area, the rock formations along the faults in the study area are broken, joints are developed, weathering is strong and loose material reserves are large. It provides dynamic conditions and material sources for geological disasters [43]. As shown in Figure 6e, on the whole, the percentage of landslides within 1000 m of the fault is the largest, and the percentage of landslides beyond 1000 m is similar. It shows that the influence range of the fault in the study area is about 1000 m, and beyond this range, the fault has little influence on the occurrence of landslides. The percentage of landslides in NRS is negatively correlated with the distance. The lower the distance, the higher the landslide density. In RS, the percentage of landslides is not strongly correlated with the distance. After the distance exceeds 1000 m, the percentage of landslides changes greatly, indicating that rainfall in the area greater than 1000 m has a greater impact on the fault, resulting in a significant change in the percentage of landslides.

Constrained by topographic factors, most of the main roads in the study area are built on the mountains, and the excavation of the road construction on the foot of the slope affects the overall stability of the nearby mountain. Moreover, the man-made effects such as slope cutting and top loading during road construction in the study area are more prominent, affecting the geomorphic features and landslide distribution along with the road and triggering many landslide hazards [17,40]. From Figure 6f, the percentage of landslides in NRS is negatively correlated with the distance, and the closer the distance, the higher the percentage of landslides, indicating that the influence range of the road in the study area is roughly around 1000 m, and has little influence on the percentage of landslides after this range. Rainfall increases the water saturation at the bottom of the slope, which in turn increases the number of landslides. It was observed that the percentage of landslides in RS at 1000 m has a more obvious increase compared with NRS, indicating that landslides are more likely to occur near the road during RS. Additionally, it can be seen that the percentage of landslides fluctuates slightly after the distance exceeds 1000 m in RS, indicating that the rainfall has an effect on the distance, and the frequent rainfall in RS further amplifies these artificial effects.

River banks gradually steepen under the lateral erosion and undercut erosion of rivers, slope stability becomes weaker and landslides are highly susceptible to occur under the influence of other factors. The degree of influence of rivers on landslides is distance dependent [57]. As can be seen from Figure 6g, the percentage of landslides is negatively correlated with the distance of the river, and the lower the distance, the higher the percentage of landslides. The percentage of landslides within 500 m of the river is the highest but decreases gradually beyond 500 m. It shows that the influence range of rivers in the study area is about 500 m, and after this range, rivers have little influence on the occurrence of landslides. It was also found that the percentage of landslides is slightly increased at greater than 2000 m, which is because there is still a large part of the study area outside the 2000 m of the river, and some landslide points that are not related to the river are counted, thus increasing the percentage. This indicates that landslide susceptibility analysis requires many sample data, and the insufficient amount of landslide data may lead to bias in some results.

Overall, the landslides in the study area are controlled by a variety of indicators and have obvious temporal and spatial distribution patterns. Temporally landslides are mostly occurring in RS. Through the statistical data, 108 landslides were found to occur in RS, accounting for 51.43%. Spatially landslides are mainly controlled by topography and lithology. Topography is the basic condition for landslides occurrence, and it is found that landslides in the study area are mostly distributed in the area between 1000 m and 26°. There were 227 landslides in the elevation range, including 139 in NRS and 88 in RS, accounting for 84.24% and 81.48%, respectively. A total of 218 landslides were in the slope range, including 139 in NRS and 79 in RS, accounting for 84.24% and 73.15%, respectively. The lithology is the intrinsic indicator and material basis for the occurrence of landslides. It was found that NRS landslides are mostly developed in [L6], and 75 landslides are developed in the region, accounting for 45.45%; RS landslides are mostly developed in [L4], [L6] and 71 landslides developed in the region, accounting for 65.74%. Other related scholars also analyzed the importance of landslide-related influencing factors and obtained results that are different from the present assessment [58,59,60,61]. The reason should be due to the complex geological environment conditions in which landslides occur and the varying degrees of the contribution of influencing factors to the occurrence of landslides, which makes it difficult to quantify accurately in the analysis process.

### 5.3. Validation of Test Points

The new landslide points were used as another method to evaluate the accuracy of the models [62]. Information was collected on four landslides that occurred in the study area at different time periods, including Shaziba [63], Baozha [64], Xintang [65] and Fujiapo landslide [66] (Figure 4g). All four landslides occurred during the RS and were triggered by rainfall in complex geological conditions.

After projecting coordinates onto the integrated landslide susceptibility zoning map, it was shown that Baozha and Shaziba landslides were located in RS high susceptibility, and Xintang and Fujiapo landslides were located in year-round high susceptibility. The accuracy of the models was further verified.

### 5.4. Prevention and Suggestions

Because socioeconomic conditions vary from region to region in China, spending on disaster prevention and mitigation varies greatly [20]. The integrated landslide susceptibility zoning map can further demonstrate the RS and NRS landslide susceptibility in different regions and refine the degree of landslide susceptibility in time. Additionally, the temporal refinement can enable the government to monitor landslide-prone areas in different time periods in a more targeted manner and help improve the accuracy of landslides monitoring. At the same time, it can also take preventive and control measures in advance for landslide-prone areas in different time periods, which can provide a scientific basis for landslide management in the study area.

According to the different distribution of landslide susceptibility in RS and NRS, prevention measures and suggestions are proposed to be combined with the integrated landslide susceptibility zoning map (Table 8). It can provide a scientific basis for landslide management in the study area.

## 6. Conclusions

This study captured the characteristic that the number of landslides in RS is significantly more than that in NRS in the Enshi area and assessed the landslide susceptibility, respectively. The results of landslide susceptibility zoning show that the distribution of landslide susceptibility in RS is significantly different from that in NRS. Additionally, a new integrated landslide susceptibility zoning method was proposed; the integrated susceptibility zoning map can better show the landslide susceptibility at different time periods. Considering the complexity and difficulty of landslide occurrence prediction, the techniques and models used for landslide susceptibility assessment require a large amount of data input. The amount of data in this study is still not large enough, which leads to bias in the calculations. It is suggested that the government can establish a perfect statistical system to keep detailed and accurate information about each landslide to provide security for related research. Although this study did not specifically assess landslide susceptibility in a particular time region, this study can serve as a useful additional reference. It is hoped that future studies can further refine the relationship between landslide susceptibility and time.

## Figures and Tables

**Table 1:** The categories of lithology in the study area.

Categories of Lithology	Category Code	Lithology
Loose soil category	[L1]	Loose soil
Layered clastic rocks category	[L2]	Weak mass–thick layered sandstone with shale and conglomerate
	[L3]	Hard–weak layered siltstone, quartz sandstone, mudstone
	[L4]	Hard–weak and thin–medium thick layered shale, siltstone, mudstone
Layered Carbonate Rocks interspersed with clastic rocks category	[L5]	Hard–relatively hard thick layered quartz sandstone, shale
	[L6]	Hard–relatively harder, limestone, siliceous shale, carbon shale
	[L7]	Hard–relatively hard and thin-thick layered shale, limestone
Layered carbonate rocks category	[L8]	Hard thick layered limestone, dolomite, breccia
	[L9]	Hard block–thick layered limestone
	[L10]	Hard thick layer–medium thick layered limestone, siliceous limestone, bioclastic limestone, dolomite

**Table 2:** Meaning of 1–9 scale method (modified by Ma [50] et al.).

Scale	Meaning
1	Equal important
3	The one is slightly more important than the other
5	The one is more important than the other
7	The one is significantly more important than the other
9	The one is extremely more important than the other
2, 4, 6, 8	The median value of the above two adjacent judgments

**Table 3:** Judgment matrix of the importance of assessment indicators.

Assessment Indicators	Judgement Matrix										*P_i_*
E (m)											
<700	1	1	4/3	2	4						0.286
700–1000		1	4/3	2	4						0.286
1000–1300			1	3/2	3						0.214
1300–1600				1	2						0.143
>1600					1						0.071
S (°)											
<10	1	1/2	1/3	1/2	1						0.111
10–18		1	3/2	1	2						0.222
18–26			1	3/2	3						0.333
26–36				1	2						0.222
>36					1						0.111
A											
Flat	1	1/4	1/4	1/3	1/2	1/2	1/4	1/3	1/3		0.039
North		1	1	4/3	2	2	1	4/3	4/3		0.154
Northeast			1	4/3	2	2	1	4/3	4/3		0.154
East				1	3/2	3/2	3/4	1	1		0.116
Southeast					1	1	1/2	2/3	2/3		0.077
South						1	1/2	2/3	2/3		0.077
Southwest							1	4/3	4/3		0.154
West								1	1		0.115
Northwest									1		0.115
DF (m)											
<1000	1	5/4	5/3	5/2	5						0.333
1000–2000		1	4/3	2	4						0.267
2000–3000			1	3/2	3						0.200
3000–4000				1	2						0.133
>4000					1						0.067
DRo (m)											
<1000	1	5/4	5/3	5/2	5						0.333
1000–2000		1	4/3	2	4						0.267
2000–3000			1	3/2	3						0.200
3000–4000				1	2						0.133
>4000					1						0.067
Rainfall in RS (mm)											
<224	1	5/4	5/3	5/2	5						0.067
224–239		1	4/3	2	4						0.133
239–254			1	3/2	3						0.200
254–269				1	2						0.267
>269					1						0.333
Rainfall in NRS (mm)											
<91	1	5/4	5/3	5/2	5						0.067
91–99		1	4/3	2	4						0.133
99–107			1	3/2	3						0.200
107–116				1	2						0.267
>116					1						0.333
L											
L10	1	1/5	1/4	1/4	1/3	1/3	1/3	1/2	1	1/2	0.035
L1		1	1	5/4	5/3	5/3	5/3	5/2	5	5/2	0.172
L2			1	5/4	5/3	5/3	5/3	5/2	5	5/2	0.172
L3				1	4/3	4/3	4/3	2	4	2	0.138
L4					1	1	1	3/2	3	3/2	0.103
L5						1	1	3/2	3	3/2	0.103
L6							1	3/2	3	3/2	0.103
L7								1	2	1	0.069
L8									1	2/1	0.035
L9										1	0.069
DRi (m)											
<500	1	5/4	5/3	5/2	5						0.333
500–1000		1	4/3	2	4						0.267
1000–1500			1	3/2	3						0.200
1500–2000				1	2						0.133
>2000					1						0.067

**Table 4:** Judgment matrix of the weight of assessment indicators during RNS.

Assessment Indicators	E	S	A	DF	DRi	Rainfall in NRS	DRo	L
E	1	5/2	5/3	5	1	5/4	5/2	5/6
S		1	2/3	2	2/5	1/2	1	1/3
A			1	3	3/5	3/4	3/2	1/2
DF				1	1/5	1/4	1/2	1/6
DRi					1	5/4	5/2	5/6
Rainfall in NRS						1	2	2/3
DRo							1	1/3
L								1
*Wi*	0.177	0.071	0.107	0.036	0.179	0.143	0.071	0.214

**Table 5:** Judgment matrix of the weight of assessment indicators during RS.

Assessment Indicators	E	D	S	DF	DRi	Rainfall in RS	DRo	L
E	1	3	3/4	3	1	3	3/2	1/2
S		1	1/4	1	1/3	1	1/2	1/6
A			1	4	4/3	4	2	2/3
DF				1	1/3	1	1/2	1/6
DRi					1	3	3/2	1/2
Rainfall in RS						1	1/2	1/6
DRo							1	1/3
L								1
*Wi*	0.143	0.048	0.191	0.048	0.143	0.048	0.095	0.286

**Table 6:** Information quantity for each assessment indicators.

Assessment Indicators	*N_j_* (NRS)	*N_j_*/*N* (NRS)	*N_j_* (RS)	*N_j_*/*N* (RS)	*S_j_*	*S_j_*/*S*	*I_ij_* (NRS)	*I_ij_* (RS)
E (m)								
<700	59	0.358	33	0.333	100,655	0.205	0.556	0.486
700–1000	60	0.364	33	0.333	140,040	0.285	0.243	0.156
1000–1300	30	0.182	22	0.222	117,615	0.240	−0.276	−0.075
1300–1600	14	0.085	11	0.111	79,967	0.163	−0.653	−0.383
>1600	2	0.012	0	0.000	52,658	0.107	−2.182	0.00
S (°)								
<10	35	0.212	23	0.232	122,805	0.250	−0.165	−0.074
10–18	67	0.406	28	0.283	156,153	0.318	0.244	−0.118
18–26	37	0.224	28	0.283	114,337	0.233	−0.038	0.194
26–36	17	0.103	14	0.141	70,368	0.143	−0.330	−0.013
>36	9	0.055	6	0.061	27,272	0.056	−0.018	0.088
A								
Flat	0	0.000	0	0.000	590	0.001	0.000	0.000
North	15	0.092	13	0.131	51,089	0.104	−0.129	0.232
Northeast	16	0.098	14	0.141	53,736	0.110	−0.115	0.256
East	31	0.189	17	0.172	75,192	0.153	0.210	0.115
Southeast	23	0.140	10	0.101	69,224	0.141	−0.006	−0.334
South	14	0.085	7	0.071	50,082	0.100	−0.178	−0.367
Southwest	20	0.122	14	0.141	54,518	0.111	0.095	0.242
West	24	0.146	13	0.131	71,413	0.146	0.006	−0.103
Northwest	21	0.128	11	0.111	65,091	0.133	−0.035	−0.177
DF (m)								
<1000	42	0.255	25	0.232	7442	0.215	0.169	0.074
1000–2000	28	0.170	12	0.130	6576	0.190	−0.113	−0.383
2000–3000	28	0.170	20	0.185	5748	0.166	0.022	0.110
3000–4000	26	0.158	24	0.222	4626	0.134	0.165	0.509
>4000	41	0.249	25	0.232	10,226	0.295	−0.173	−0.244
DRo (m)								
<1000	65	0.394	52	0.482	10,652	0.308	0.247	0.448
1000–2000	31	0.188	12	0.111	7312	0.211	−0.117	−0.643
2000–3000	25	0.152	17	0.157	5251	0.152	−0.001	0.037
3000–4000	19	0.115	13	0.120	3806	0.110	0.047	0.091
>4000	25	0.152	14	0.130	7597	0.220	−0.371	−0.527
Rainfall in RS (mm)								
<224			10	0.093	3186	0.092		0.007
224–239			50	0.463	14,198	0.410		0.121
239–254			29	0.269	10,636	0.307		−0.135
254–269			18	0.167	5239	0.151		0.097
>269			1	0.009	1359	0.039		−1.441
Rainfall in NRS (mm)								
<91	81	0.491			9,695	0.280	0.561	
91–99	42	0.255			10,933	0.316	−0.216	
99–107	21	0.127			7152	0.207	−0.484	
107–116	20	0.121			6133	0.177	−0.380	
>116	1	0.006			705	0.020	−1.207	
L								
L1	0	0.000	0	0.000	171	0.005	0.000	0.000
L2	1	0.006	9	0.083	1327	0.038	−1.837	0.777
L3	24	0.146	7	0.065	1341	0.039	1.324	0.516
L4	23	0.139	22	0.204	5270	0.152	−0.088	0.292
L5	8	0.049	5	0.046	1306	0.038	0.252	0.206
L6	75	0.455	46	0.426	12,175	0.352	0.256	0.191
L7	4	0.024	1	0.009	329	0.010	0.935	−0.021
L8	10	0.061	5	0.046	4553	0.132	−0.775	−1.044
L9	18	0.109	9	0.083	4333	0.125	−0.138	−0.408
L10	2	0.012	4	0.037	3813	0.110	−2.208	−1.090
DRi (m)								
<500	74	0.449	51	0.472	9011	0.206	0.544	0.596
500–1000	50	0.303	22	0.204	7615	0.220	0.320	−0.077
1000–1500	15	0.091	13	0.120	5797	0.168	−0.611	−0.330
1500–2000	8	0.049	7	0.065	4029	0.116	−0.875	−0.586
>2000	18	0.109	15	0.139	8166	0.236	−0.771	−0.530

**Table 7:** Principles of map integration.

Integrate Susceptibility	RS Susceptibility	NRS Susceptibility
Year-round high susceptibility	High and very high susceptibility	High and very high susceptibility
RS high susceptibility	High and very high susceptibility	Medium, low and very low susceptibility
NRS high susceptibility	Medium, low and very low susceptibility	High and very high susceptibility
Year-round low susceptibility	Low and very low susceptibility	Low and very low susceptibility

**Table 8:** Prevention measures and suggestions for the study area.

Integrate Susceptibility	Prevention Measures	Suggestions
Year-round high susceptibility	(1) Detailed investigation and management of landslides according to their characteristics, such as construction of retaining walls, grouting reinforcement, etc. (2) Establish monitoring stations for landslides that may produce hazards and monitor the landslides at times.	(1) Follow the principle of prevention as the main focus, and combine it with management. (2) Vigorously promote the knowledge of landslides prevention and control to enhance the ability of the whole society to resist hazards. (3) Improve landslides monitoring network system construction, according to the year-round high susceptibility > NRS high susceptibility > RS high susceptibility > year-round low susceptibility gradually improved. (4) Protect the local vegetation, actively plant trees and prohibit indiscriminate logging. (5) Strictly implement the principle of safe construction and conduct safety assessment before carrying out human engineering activities.
RS high susceptibility	(1) Survey landslides in the region before the RS and provide early warning to nearby residents. (2) Increasing investment in landslides management during the RS and monitoring or managing landslides that may produce hazards. (3) Surface and underground drains are constructed in the area to reduce infiltration of atmospheric precipitation and recharge of groundwater.	
NRS high susceptibility	(1) Reminding the residents to pay attention to the landslides deformation and report any problems in time. (2) Monitor or manage landslides in the region that may produce hazards.	
Year-round low susceptibility	/	

## Data Availability

Not applicable.

## Supplementary Materials

The following supporting information can be downloaded at: https://www.mdpi.com/article/10.3390/ijerph19159412/s1. Table S1. Landslides occurrence of time and coordinate information. Table S2: Rainfall information for the study area.
