# Supplementary material for: Refined Zoning of Landslide Susceptibility: A Case Study in Enshi County, Hubei, China
Source: Int J Environ Res Public Health. 2022 Aug 1;19(15):9412. doi: 10.3390/ijerph19159412 (PMC9368755; doi:10.3390/ijerph19159412)
Supplement: Supplementary file 1 [file ijerph-19-09412-s001.zip › ijerph-1788533-supplementary.pdf]

Supplementary Materials：Catalogue list of landslides

Catalogue list of landslides：

The information of a total of 108 rainfall landslide data points in the study area was collected by means of field survey and historical data compilation.The time and coordinate information of landslide occurrence are shown in Table S1, and the information on rainfall associated with landslides is shown in Table S2.

Coordinate system： China Geodetic Coordinate System 2000 (CGCS2000).

Table S1. Landslides occurrence of time and coordinate information.

Serial No.	Occurrence time	X	Y
1	1997.06.12	109.2490005	30.5841999
2	1970.07.10	109.2470016	30.5797997
3	2005.07.15	109.2440033	30.5788002
4	2005.08.13	109.1780014	30.4976997
5	1997.07.20	109.197998	30.4354992
6	1997.07.18	109.1900024	30.4228992
7	1998.07.01	109.1920013	30.4237995
8	2004.07.18	109.2360001	30.4125004
9	2005.07.09	109.1949997	30.3924999
10	1997.06.12	109.4290009	30.4109001
11	1983.07.03	109.3889999	30.3141994
12	1990.07.13	109.322998	30.2961998
13	2001.08.17	109.3519974	30.1884003
14	1988.06.13	109.2369995	30.1420994
15	2001.08.13	109.1380005	30.0590992
16	2000.07.24	109.237999	30.0632992
17	1978.07.08	109.2330017	30.0634003
18	1978.07.06	109.2450027	30.0438004
19	2004.07.15	109.2450027	30.0287991
20	1978.07.23	109.189003	30.0154991
21	2000.08.15	109.3519974	30.1275005
22	1993.06.07	109.3430023	30.1016006
23	1980.06.27	109.4120026	30.0881004
24	1980.06.23	109.4420013	30.0960999
25	2006.06.29	109.4120026	30.1252003
26	2006.06.20	109.4290009	30.1254005
27	2006.07.11	109.4410019	30.1434994
28	2006.06.20	109.4319992	30.1499996
29	1987.07.07	109.4499969	30.1662998
30	1983.08.15	109.4609985	30.1676998
31	2005.06.07	109.4929962	30.1480999
32	2004.07.14	109.4759979	30.1380005

Serial No.	Occurrence time	X	Y
33	2000.08.17	109.5090027	30.1480999
34	1978.06.14	109.5059967	30.1578999
35	1996.06.10	109.5080032	30.1615009
36	1983.08.15	109.5019989	30.1800995
37	1997.07.16	109.4950027	30.1786995
38	2004.06.20	109.5159988	30.1919994
39	2005.06.06	109.4960022	30.1935005
40	1976.06.18	109.4430008	30.1900997
41	1997.07.16	109.4089966	30.1931
42	1997.07.15	109.612999	30.2252998
43	1997.07.15	109.6029968	30.2609997
44	1997.07.16	109.5999985	30.2651997
45	1997.07.16	109.6460037	30.1826
46	1997.07.11	109.6600037	30.2150002
47	1997.07.15	109.6940002	30.2283993
48	1997.07.16	109.697998	30.2259007
49	1997.07.16	109.711998	30.2264004
50	1997.07.16	109.7009964	30.2210999
51	1997.07.16	109.6999969	30.2136993
52	1997.07.20	109.7040024	30.2043991
53	1997.07.16	109.8270035	30.2017994
54	1997.07.16	109.8059998	30.2285995
55	1997.07.16	109.6790009	30.2514992
56	1997.07.16	109.6900024	30.2584
57	1997.07.16	109.7529984	30.2637997
58	1997.07.16	109.7509995	30.2586002
59	1997.07.16	109.7519989	30.2530003
60	1997.07.16	109.7730026	30.2987003
61	1997.07.16	109.7860031	30.2936001
62	1997.07.19	109.7610016	30.2866001
63	1997.07.19	109.7679977	30.2812996
64	1997.07.10	109.8150024	30.2889996
65	1997.07.16	109.8420029	30.2695007
66	1983.06.04	109.9720001	30.1382999
67	1997.07.16	109.8949966	30.2793007
68	1983.06.30	109.9469986	30.2894001
69	2001.08.15	109.9339981	30.2749004
70	1980.08.03	109.935997	30.2744007
71	1991.08.16	109.9219971	30.3048992
72	1980.07.25	109.9229965	30.3437996
73	1960.07.16	109.7429962	30.3444004
74	1980.08.11	109.8610001	30.4143009
75	2005.07.10	109.8899994	30.4211006

Serial No.	Occurrence time	X	Y
76	1980.07.28	109.7300034	30.4503994
77	1977.07.03	109.7350006	30.4487
78	1982.07.02	109.6809998	30.4701004
79	1985.07.19	109.6910019	30.4304008
80	1980.06.23	109.6289978	30.4239998
81	1997.06.15	109.612999	30.4113007
82	1998.07.15	109.6220016	30.3857002
83	1997.07.16	109.6159973	30.3710995
84	1997.07.16	109.4990005	30.6138
85	2000.08.07	109.473999	30.5681
86	1997.07.16	109.5459976	30.5356007
87	1997.07.16	109.5500031	30.5291004
88	1997.07.16	109.4950027	30.4964008
89	2005.07.03	109.4169998	30.4748993
90	2005.07.03	109.4599991	30.4594002
91	2000.08.30	109.4540024	30.4554005
92	1997.07.16	109.4789963	30.4540997
93	1997.08.16	109.4720001	30.4435005
94	1997.07.16	109.4860001	30.4748001
95	2000.08.15	109.5110016	30.4622002
96	2000.06.10	109.4260025	30.4302998
97	1997.08.13	109.4580002	30.4276009
98	2000.07.15	109.5459976	30.4073009
99	1997.07.10	109.5070038	30.3659992
100	1997.07.16	109.4919968	30.3456001
101	1997.06.12	109.5070038	30.3239994
102	1997.07.16	109.4189987	30.3055
103	1976.06.15	109.4550018	30.2831993
104	2001.08.17	109.4970016	30.281601
105	1997.07.12	109.4749985	30.2735004
106	1991.08.17	109.4759979	30.2693005
107	1997.07.16	109.4759979	30.2523994
108	2005.07.12	109.7529984	30.2539997

Table S2. Rainfall information for the study area.

Time	Precipitation (mm*0.1)	Time	Precipitation (mm*0.1)	Time	Precipitation (mm*0.1)
1960.07.07	884	1985.07.13	226	2000.07.20	0
1960.07.08	20	1985.07.14	277	2000.07.21	0
1960.07.09	17	1985.07.15	0	2000.07.22	0
1960.07.10	0	1985.07.16	25	2000.07.23	0
1960.07.11	167	1985.07.17	2	2000.07.24	0
1960.07.12	49	1985.07.18	0	2000.08.02	144

Time	Precipitation (mm*0.1)	Time	Precipitation (mm*0.1)	Time	Precipitation (mm*0.1)
1960.07.13	8	1985.07.19	0	2000.08.03	739
1960.07.14	16	1987.07.01	182	2000.08.04	25
1960.07.15	152	1987.07.02	58	2000.08.05	28
1960.07.16	2	1987.07.03	58	2000.08.06	79
1970.07.03	405	1987.07.04	74	2000.08.07	58
1970.07.04	0	1987.07.05	322	2000.08.27	23
1970.07.05	22	1987.07.06	226	2000.08.28	0
1970.07.06	124	1987.07.07	173	2000.08.29	0
1970.07.07	455	1988.06.10	460	2000.08.30	0
1970.07.08	26	1988.06.11	833	2000.10.10	54
1970.07.09	57	1988.06.12	111	2000.10.11	200
1970.07.10	82	1988.06.13	1	2000.10.12	205
1970.07.11	2	1990.07.10	107	2000.10.13	44
1970.07.12	169	1990.07.11	85	2000.10.14	5
1976.06.06	15	1990.07.12	0	2000.10.15	0
1976.06.07	13	1990.07.13	0	2000.10.24	103
1976.06.08	0	1991.04.12	17	2000.10.25	64
1976.06.09	64	1991.04.13	16	2000.10.26	369
1976.06.10	0	1991.04.14	3	2000.10.27	358
1976.06.11	0	1991.04.15	19	2001.08.08	188
1976.06.12	0	1991.04.16	142	2001.08.09	28
1976.06.13	0	1993.06.03	119	2001.08.10	17
1976.06.14	2	1993.06.04	64	2001.08.11	0
1976.06.15	42	1993.06.05	0	2001.08.12	9
1976.06.16	40	1993.06.06	0	2001.08.13	0
1976.06.17	241	1993.06.07	0	2004.06.18	356
1976.06.18	18	1996.05.31	25	2004.06.19	0
1976.06.19	7	1996.06.01	40	2004.06.20	0
1976.06.20	1	1996.06.02	72	2004.06.21	0
1977.07.02	0	1996.06.03	580	2004.06.22	0
1977.07.03	247	1996.06.04	290	2004.06.23	33
1978.06.10	0	1996.06.05	184	2004.06.24	4
1978.06.11	69	1996.06.06	24	2004.06.25	0
1978.06.12	413	1996.06.07	240	2004.06.26	0
1978.06.13	180	1996.06.08	380	2004.06.27	0
1978.06.14	0	1996.06.09	10	2004.06.28	0
1978.07.04	2	1996.06.10	11	2004.06.29	0
1978.07.05	439	1997.06.05	25	2004.06.30	0
1978.07.06	0	1997.06.06	184	2004.07.01	232
1978.07.07	0	1997.06.07	390	2004.07.02	0
1978.07.08	0	1997.06.08	255	2004.07.03	1
1978.07.22	0	1997.06.09	296	2004.07.04	0

Time	Precipitation (mm*0.1)	Time	Precipitation (mm*0.1)	Time	Precipitation (mm*0.1)
1978.07.23	253	1997.06.10	0	2004.07.05	0
1980.06.16	105	1997.06.11	0	2004.07.06	3
1980.06.17	2275	1997.06.12	5	2004.07.07	75
1980.06.18	133	1997.06.13	0	2004.07.08	0
1980.06.19	277	1997.06.14	0	2004.07.09	0
1980.06.20	12	1997.06.15	0	2004.07.10	447
1980.06.21	56	1997.06.29	39	2004.07.11	9
1980.06.22	11	1997.06.30	450	2004.07.12	367
1980.06.23	28	1997.07.01	66	2004.07.13	5
1980.06.24	147	1997.07.02	6	2004.07.14	0
1980.06.25	337	1997.07.03	0	2004.07.15	95
1980.06.26	116	1997.07.04	188	2004.07.16	79
1980.06.27	0	1997.07.05	73	2004.07.17	138
1980.07.16	244	1997.07.06	141	2004.07.18	4
1980.07.17	417	1997.07.07	323	2004.12.22	128
1980.07.18	90	1997.07.08	0	2004.12.23	2
1980.07.19	690	1997.07.09	0	2004.12.24	4
1980.07.20	2	1997.07.10	0	2004.12.25	0
1980.07.21	0	1997.07.11	0	2004.12.26	12
1980.07.22	0	1997.07.12	68	2004.12.27	3
1980.07.23	0	1997.07.13	408	2004.12.28	0
1980.07.24	0	1997.07.14	1350	2004.12.29	2
1980.07.25	0	1997.07.15	327	2004.12.30	3
1980.07.26	0	1997.07.16	1415	2004.12.31	0
1980.07.27	0	1997.07.17	0	2005.01.01	0
1980.07.28	0	1997.07.18	122	2005.01.02	0
1980.07.29	0	1997.07.19	617	2005.01.03	0
1980.07.30	73	1997.07.20	1	2005.01.04	0
1980.07.31	184	1997.08.08	475	2005.01.05	0
1980.08.01	772	1997.08.09	0	2005.01.06	0
1980.08.02	57	1997.08.10	0	2005.06.04	22
1980.08.03	1046	1997.08.11	0	2005.06.05	306
1980.08.04	886	1997.08.12	0	2005.06.06	363
1980.08.05	0	1997.08.13	0	2005.06.07	0
1980.08.06	0	1998.06.28	351	2005.06.25	101
1980.08.07	6	1998.06.29	395	2005.06.26	0
1980.08.08	1	1998.06.30	146	2005.06.27	0
1980.08.09	0	1998.07.01	157	2005.06.28	0
1980.08.10	367	1998.07.02	714	2005.06.29	0
1980.08.11	58	1998.07.03	37	2005.06.30	0
1982.06.27	329	1998.07.04	548	2005.07.01	0
1982.06.28	20	1998.07.05	4	2005.07.02	0

Time	Precipitation (mm*0.1)	Time	Precipitation (mm*0.1)	Time	Precipitation (mm*0.1)
1982.06.29	0	1998.07.06	1	2005.07.03	0
1982.06.30	3	1998.07.07	3	2005.07.04	0
1982.07.01	0	1998.07.08	7	2005.07.05	0
1982.07.02	0	1998.07.09	2	2005.07.06	0
1983.06.01	337	1998.07.10	0	2005.07.07	0
1983.06.02	0	1998.07.11	82	2005.07.08	333
1983.06.03	0	1998.07.12	106	2005.07.09	1043
1983.06.04	0	1998.07.13	9	2005.07.10	999
1983.06.22	51	1998.07.14	1	2005.07.11	1
1983.06.23	55	1998.07.15	22	2005.07.12	0
1983.06.24	78	2000.04.10	35	2005.07.13	0
1983.06.25	321	2000.04.11	10	2005.07.14	0
1983.06.26	576	2000.04.12	0	2005.07.15	57
1983.06.27	55	2000.04.13	0	2006.06.20	43
1983.06.28	195	2000.04.14	15	2006.06.21	1
1983.06.29	506	2000.06.03	153	2006.06.22	0
1983.06.30	244	2000.06.04	261	2006.06.23	2
1983.07.01	295	2000.06.05	144	2006.06.24	1
1983.07.02	0	2000.06.06	84	2006.06.25	1
1983.07.03	0	2000.06.07	1	2006.06.26	7
1983.08.08	143	2000.06.08	280	2006.06.27	3
1983.08.09	8	2000.06.09	1	2006.06.28	0
1983.08.10	112	2000.06.10	45	2006.06.29	23
1983.08.11	4	2000.07.14	68	2006.07.10	2
1983.08.12	61	2000.07.15	84	2006.07.11	20
1983.08.13	0	2000.07.16	25	2006.07.12	1
1983.08.14	45	2000.07.17	0		
1983.08.15	0	2000.07.18	0		
